# Nonalcoholic Fatty Liver Disease Cirrhosis: A Review of Its Epidemiology, Risk Factors, Clinical Presentation, Diagnosis, Management, and Prognosis

**DOI:** 10.1155/2018/2784537

**Published:** 2018-07-02

**Authors:** Bei Li, Chuan Zhang, Yu-Tao Zhan

**Affiliations:** Department of Gastroenterology, Beijing Tongren Hospital, Capital Medical University, Beijing 100730, China

## Abstract

Cirrhosis is the common end stage of a number of chronic liver conditions and a significant cause of morbidity and mortality. With the growing epidemic of obesity and metabolic syndrome, nonalcoholic fatty liver disease (NAFLD) has become the most common cause of chronic liver disease worldwide and will become one of the leading causes of cirrhosis. Increased awareness and understanding of NAFLD cirrhosis are essential. To date, there has been no published systematic review on NAFLD cirrhosis. Thus, this article reviews recent studies on the epidemiology, risk factors, clinical presentation, diagnosis, management, and prognosis of NAFLD cirrhosis.

## 1. Introduction

Cirrhosis is the end stage of a wide number of chronic liver conditions that share common features of necroinflammation, fibrosis, and regenerative nodules, which modify the normal liver structure to reduce its functional mass and alter the vascular architecture [1]. Cirrhosis has become a major public health problem and a significant cause of morbidity and mortality [2]. It is the 13th most common cause of mortality worldwide [3]. Global cirrhosis deaths have increased from 1.54% of all deaths in 1980 to 1.95% in 2010 [4], causing more than one million deaths each year [5]. The most common primary etiologies for cirrhosis are chronic hepatitis B, alcoholic liver disease, chronic hepatitis C, and nonalcoholic fatty liver disease (NAFLD) [2]. Chronic hepatitis B is the most common cause of cirrhosis in most parts of Asia and sub-Saharan Africa [4], whereas alcoholic liver disease and chronic hepatitis C are the main causes in most developed countries. In recent years, with the rising incidence of obesity, NAFLD has become one of the leading causes of cirrhosis in some countries [6]. By 2020, the number of individuals with NAFLD cirrhosis is predicted to exceed that of those with hepatitis B- and C-related cirrhosis, and NAFLD cirrhosis will become the leading indication for liver transplantation [7].

## 2. Epidemiology

With the ongoing epidemic of obesity and metabolic syndrome, NAFLD has become the most common cause of chronic liver disease worldwide [8]. The global prevalence of NAFLD was estimated to be about 24% [9]. Cirrhosis is an important factor for liver-related morbidity and mortality in patients with NAFLD [10]. However, we still do not have a detailed understanding on how often NAFLD cirrhosis occurs. Existing studies with different study objects, diagnostic methods, and other variable parameters showed the inconsistent epidemiological results of NAFLD cirrhosis.

### 2.1. General Population Study

Kabbany et al. analyzed the National Health and Nutrition Examination Survey (USA) data between 2009 and 2012. Cirrhosis was diagnosed by an AST to platelet ratio index >2 and abnormal liver function tests. NAFLD cirrhosis was defined as cirrhosis that presented with at least one of the following: obesity, diabetes, insulin resistance, and metabolic syndrome. They reported that the prevalence of NAFLD cirrhosis was 0.178% [11]. Fung et al. performed a prospective cross-sectional study of 2493 volunteers recruited from the general population and the Red Cross Transfusion Center in Hong Kong (China). Cirrhosis was diagnosed by transient elastography (TE). They found that the incidence of NAFLD cirrhosis was 0.17 % [12].

### 2.2. Diseases or Morbidity Patients Study

A study on 1799 patients with type 2 diabetes (T2DM) showed that the prevalence of NAFLD cirrhosis diagnosed by TE was 11.2% [13]. A review of 16 individual studies of 2,956 patients with severe obesity revealed that 5.8% of patients have NAFLD cirrhosis [8]. Those studies suggested that patients with T2DM or severe obesity have high incidence of NAFLD cirrhosis [14].

### 2.3. Hospitalized Patients with Cirrhosis Study

Xiong et al. performed a retrospective study of 1,582 patients with cirrhosis at Daping Hospital (China). Cirrhosis was diagnosed based on clinical symptoms, imaging data, and/or histological findings. This study found that the prevalence of NAFLD cirrhosis was 1.9% [15]. Michitaka et al. analyzed data from 33,379 patients with cirrhosis at 58 hospitals including all university and other major hospitals in Japan. Cirrhosis was diagnosed by autopsy, laparoscopy or abdominal imaging, laboratory findings, and clinical findings compatible with cirrhosis. This analysis showed that NAFLD cirrhosis constituted 2.1% of all cases of cirrhosis [16]. Karageorgos et al. studied 812 cases of cirrhosis from a liver disease center (Greece). The diagnosis of cirrhosis was confirmed by liver biopsy in compensated cirrhosis and clinical evidence in decompensated cirrhosis. They found that NAFLD cirrhosis constituted 15.5% of all cases of cirrhosis [17]. Hsiang et al. reported a retrospective study from a secondary care hospital in South Auckland (New Zealand). The diagnosis of cirrhosis was based on clinical, biochemical, histological, transient elastography, or radiological evidence accompanied by clinical signs of cirrhosis. The author found that NAFLD cirrhosis was prevalent in 16.4% of cirrhotic patients [18]. Those studies suggested that the prevalence of NAFLD cirrhosis is relatively lower in hospitalized patients with cirrhosis.

### 2.4. Liver Transplant Patient Studies

One study from the Nordic Liver Transplant between 2011 and 2015 reported that NASH cirrhosis was about 6.1% of adult patients (91/1476) listed for liver transplantation [19]. Another study from United Network for Organ Sharing database showed that NASH cirrhosis accounts for 5% of all young US patients listed for liver transplantation [20], and NASH cirrhosis increased from 1% to 16% from 2002 to 2016. The analysis of data from the Organ Procurement and Transplantation Network (OPTN) database from 2000 to 2014 also supported the increased tendency of NASH cirrhosis over time with an increase of 55.4% between 2016 and 2030 [21].

## 3. Risk Factor

### 3.1. Histological Subtype

Histological subtype is the greatest risk factor for the progression of NAFLD to cirrhosis. NAFLD has been divided into two main histological subtypes: nonalcoholic fatty liver (NAFL) and nonalcoholic steatohepatitis (NASH) [22]. The incidence of progression to cirrhosis is higher in NASH than in NAFL. A longitudinal study with a mean of 15.6 years of follow-up showed that only 1% of patients with NAFL developed cirrhosis, whereas 11% of those with NASH developed cirrhosis [23]. Moreover, NASH progressed more rapidly to cirrhosis. The annual fibrosis progression rate in patients with NASH was 0.14 stages, compared with 0.07 stages in patients with NAFL [24].

### 3.2. Metabolic Factors

Many studies suggested that diabetes is the strongest metabolic factor of progression of NAFLD to cirrhosis [25]. Porepa et al. used administrative health databases in Ontario (Canada) (1994–2006) to perform a population-based matched retrospective cohort study. 438,069 individuals with newly diagnosed diabetes were matched to 2,059,708 individuals without diabetes. After a median follow-up duration of 6.4 years, 1,119 (3.71%) patients with diabetes developed cirrhosis and 1,896 (1.34%) individuals without diabetes developed cirrhosis [26]. Nderitu et al. examined 509,436 participants from the Swedish Apolipoprotein Mortality Risk (AMORIS) cohort between 1985 and 1996 and found that 2,775 participants developed cirrhosis; diabetes and high blood glucose were associated with cirrhosis independent of obesity [27]. Other metabolic factors, including hyperlipidemia, obesity, and hypertension, were also important risk factors for NAFLD cirrhosis.

### 3.3. Genetic Polymorphisms

Genetic factors are believed to contribute to 30%–50% of the risk for high-prevalence diseases, such as obesity, T2DM, cardiovascular disease (CVD), and cirrhosis [28]. Genome-wide association studies (GWAS) and candidate gene studies have contributed greatly to our understanding of the genetic contribution to NAFLD progression. GWAS studies have identified some of the genetic variants associated with NAFLD progression. Among the loci identified, the nonsynonymous single-nucleotide polymorphism (SNP) in PNPLA3 (rs738409 c.444 C4G, p.Ile148Met), patatin-like phospholipase domain containing 3, has been validated across multiple patient cohorts. Notably, presence of this SNP has been robustly associated with the development of NAFLD cirrhosis [29]. One study of over 1000 individuals with biopsy-proven NAFLD demonstrated that the SNP in transmembrane 6 superfamily member 2 (rs58542926 c.449 C>T, p.Glu167Lys) was associated with increased risk for advanced fibrosis independent of gender, age at biopsy, BMI, T2DM, and PNPLA3 rs738409 genotype [30].

### 3.4. Age

In a retrospective cohort study from the United Kingdom, 351 patients with biopsy-proven NAFLD were divided into an older (≥60), a middle-aged (50 to 60), and a younger (≤50) group. Cirrhotic patients were significantly older than noncirrhotic patients. Older patients had significantly more risk factors, including hypertension, obesity, diabetes, and hyperlipidemia [31]. In a cross-sectional multicenter study from the United States, 796 patients with biopsy-proven NAFLD were classified into the elderly patients group (≥65) and the nonelderly patients group (18 to 65). Elderly patients with NAFLD had significantly higher rates of advanced fibrosis than nonelderly patients with NAFLD. Moreover, the elderly patients did not have more risk factors such as diabetes or insulin resistance [32]. However, the association between age and cirrhosis in NAFLD may be related to the duration of disease rather than the age itself [33].

### 3.5. Other Factors

Other risk factors for progression to cirrhosis in patients with NAFLD include gender, ethnicity, and family history of metabolic traits. Data on gender differences in the development of cirrhosis in patients with NAFLD are discordant [34]. A longitudinal study of patients with NAFLD found that gender was not an independent risk factor for the progression of fibrosis. A few studies suggested that male gender is a strong independent risk factor for fibrosis. Some studies showed that the risk of advanced fibrosis is higher in females than in males. Although the risk of NASH was higher in Hispanics and lower in Blacks than Whites, the proportion of patients with significant fibrosis did not significantly differ among racial or ethnic groups in United States. Thus, ethnicity is not a risk factor for the development of cirrhosis in patients with NAFLD [35]. A recent study showed that 68.8% (779/1133) of patients with NASH cirrhosis have the family history of metabolic traits, and those patients have increased risk of cirrhosis diagnosis at an early age of <45 years. Those results suggested that the family history of metabolic traits is a risk factor for cirrhosis and associated with early age at diagnosis of NAFLD cirrhosis [36].

## 4. Clinical and Liver Function Features

The clinical presentations of NAFLD cirrhosis were analyzed from several early studies [37–39]. The majority of patients with NAFLD cirrhosis are female, older than 50 years, and frequently with obesity and/or T2DM. Patients with NAFLD cirrhosis are at risk of the same complications of cirrhosis as with any other etiology of liver disease [40]. Ascites is the first and most common clinical feature of decompensation, but occurs at a slower rate in patients with NAFLD cirrhosis than in patients with HCV cirrhosis [23]. Once ascites develops, the rate of hepatorenal syndrome in patients with NAFLD cirrhosis is similar to that in patients with HCV cirrhosis [41]. The incidences of variceal hemorrhage, hepatic encephalopathy, and hepatocellular carcinoma (HCC) were similar in NAFLD cirrhosis and HCV cirrhosis patients. Liver enzyme abnormalities are found in patients with NAFLD cirrhosis, but the degrees of liver enzyme abnormality are mild. The mean values of serum ALT, AST, AKP, and GGT are usually no more than three times of the upper limit of normal values.

## 5. Diagnosis

The diagnosis of decompensated cirrhosis is relatively easy for patients with NAFLD and is mainly based on (1) having risk factors for progression to cirrhosis, (2) excluding the other causes of cirrhosis, and (3) having cirrhosis complications. However, the diagnosis of compensated cirrhosis is difficult in patients with NAFLD due to absence of symptoms. Liver biopsy, imaging, and scoring systems for fibrosis are important methods for the diagnosis of compensated cirrhosis in patients with NAFLD.

### 5.1. Liver Biopsy

Liver biopsy represents the gold standard for diagnosis of cirrhosis. Key features of NASH, such as steatosis, ballooning, and Mallory-Denk bodies, are important histological features for the diagnosis of NAFLD cirrhosis. Steatosis is the histological feature that ties together all of the various forms of NAFLD. Steatosis may become inconspicuous in cirrhosis. However, a diagnosis of NAFLD cirrhosis can still be made if the critical features of ballooning and Mallory-Denk bodies are observed [42]. All histologic features of NASH may not be evident once it progresses to cirrhosis. Therefore, cirrhosis without features of NASH may be diagnosed as “cryptogenic cirrhosis”. T2DM and obesity are important factors for the development of NAFLD cirrhosis. In addition to excluding other known causes of cirrhosis, T2DM, obesity, and other comorbidities may help diagnose NAFLD cirrhosis without key NASH features in liver histology. Although liver biopsy is considered as the gold standard for the diagnosis of NAFLD cirrhosis, it is invasive and has several limitations, including sampling bias and complications (transient pain, anxiety and discomfort, hemorrhage, and rarely death) [43, 44].

### 5.2. Imaging Methods

In recent years, noninvasive alternative diagnostic imaging methods have been validated in comparison with liver biopsy and demonstrated good diagnostic accuracy for the diagnosis of cirrhosis. One of these techniques is TE, which produces a ‘liver stiffness measurement' (LSM) using pulsed-echo ultrasound as a surrogate marker of fibrosis. A LSM >13.0 kPa is taken as the cut-off for clinically relevant cirrhosis [45]. A meta-analysis study of 7 articles showed that the sensitivity and the specificity of TE for the diagnosis of NAFLD cirrhosis were 96.2 % and 92.2%, respectively [46]. However, the failure rate of the M probe of TE is high in patients with BMI >30 kg/m^2^ or T2DM [47]. The diagnostic accuracy for the liver fibrosis of XL probe of TE is similar to that of M probe [48]. As a result, in clinical practice, if the M probe is unreliable, the XL probe could be used [49]. Another noninvasive imaging technique for the diagnosis of cirrhosis is magnetic resonance elastography (MRE). Recent study showed that MRE has higher diagnostic accuracy in detecting liver fibrosis in patients with NAFLD compared to TE [50]. MRE may be a promising noninvasive technique for the diagnosis of NAFLD cirrhosis. The important limitation of TE and MRE is that they are not widely available.

### 5.3. Score Systems for Fibrosis

Based on demographic factors and blood tests, several scoring systems for the assessment of fibrosis or cirrhosis in NAFLD have been proposed: NAFLD fibrosis score (NFS), fibrosis-4-score (FIB-4), BARD (BMI-AST/ALT-Diabetes), enhanced liver fibrosis panel (ELF), Hepascore, Fibro Meter™, Fibro Test™, and so on [51]. NFS and FIB-4 are better than scoring systems in predicting advanced fibrosis in patients with NAFLD. NFS and FIB-4 have been recommended as screening tools to identify NAFLD patients with higher likelihoods of advanced fibrosis and/or cirrhosis in the NAFLD practice guideline from the American Association for the Study of Liver Diseases (AASLD) [52]. NFS is characterized by two cut-off values: lower cut-off value and higher cut-off value. The lower cut-off value has the highest negative predictive value to exclude advanced fibrosis. The higher cut-off value has the highest positive predictive value to identify patients with advanced fibrosis. The “gray area” between the two cut-off values is the indeterminate range [53]. FIB-4 also offers dual cut-off values as NFS: patients with score <1.45 are unlikely whereas patients with score >3.25 are likely to have advanced fibrosis.

**Figure 1:**
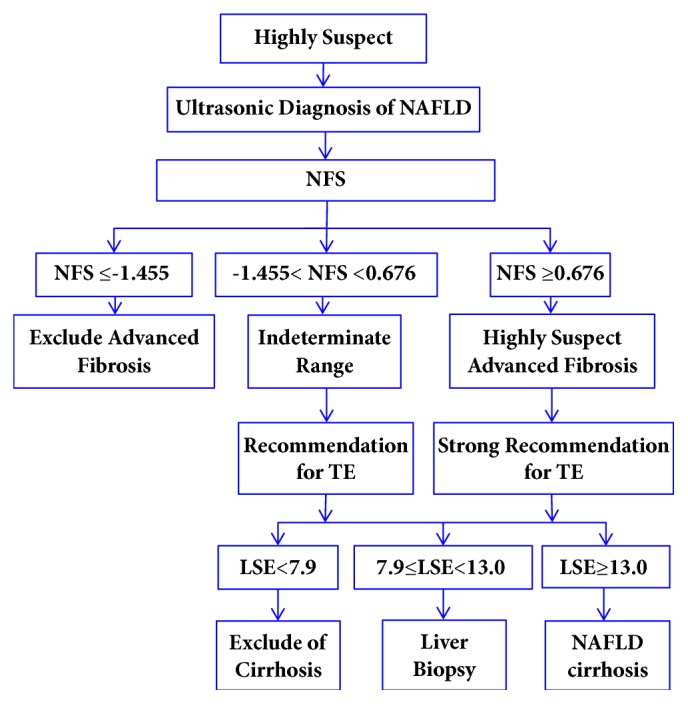
Proposal of diagnostic algorithm for classification of patients affected by NAFLD. NAFLD: nonalcoholic fatty liver disease; NFS: NAFLD fibrosis score; TE: transient elastography; LSE: liver stiffness measurement; kPa: kilopascal.

Considering the different accuracy, cost, and availability of these diagnosis methods for cirrhosis, the selection of diagnostic approach for patients with suspected NAFLD cirrhosis could be suggested as follows: (1) NFS (or FIB-4) is first used for patients with diagnosed NAFLD. (2) Cirrhosis in patients with a NFS below the lower cut-off level can be excluded. Patients with a NFS above the indeterminate range or higher cut-off level require further diagnostic testing with TE. (3) Cirrhosis in patients with a TE < 7.9 kPa can be excluded. Patients with a TE 7.9~13.0 kPa should consider liver biopsy. Patients with a TE >13.0 kPa are diagnosed as cirrhosis. A proposal of diagnostic algorithm is illustrated in Figure 1 (modified according to [45, 53]).

## 6. Management

Obesity is of great prognostic relevance to patients with cirrhosis, and weight loss is important in patients with NAFLD cirrhosis. However, weight loss should not be recommended in patients with decompensated end-stage liver disease due to the risk of protein calorie malnutrition [54]. Antifibrotic therapy is an important strategy for the prevention and reversion of NAFLD cirrhosis. Emerging drugs including activator of farnesoid X receptor (Obeticholic acid), antagonist of C-C chemokine receptors type 2 and 5 (Cenicriviroc), and inhibitor of apoptosis signaling kinase-1 (Selonsertib) have been confirmed to have antifibrotic effect and will be expected to be developed as potential therapy for NAFLD cirrhosis [55]. Alcohol is a confirmed factor for liver injury. Alcohol should be prohibited in patients with NAFLD cirrhosis. Other conditions enhancing the development of cirrhosis in patients with NAFLD include diabetes, hyperlipidemia, and hypertension, which should be screened for and treated. The prevention, screening, and treatment of CVD and cirrhosis complications are critical for the prognosis of NAFLD cirrhosis. Liver transplant is an effective treatment for end-stage liver disease in patients with NAFLD cirrhosis.

### 6.1. Surveillance and Prevention of Cardiovascular Disease

Patients with NAFLD cirrhosis have a high prevalence of CVD. Careful attention should be paid to the surveillance of CVD. Noninvasive functional cardiac testing is recommended in patients with NASH cirrhosis, with progression to invasive tests when noninvasive testing is abnormal or inconclusive [54]. Hyperlipidemia is an important factor for the development of CVD. Statins, as drugs for lipid-reduction, are recommended for the prevention of CVD in patients with NAFLD cirrhosis who meet criteria based on current recommendations, but they should be avoided in patients with decompensated cirrhosis [52].

### 6.2. Screening and Management of Gastroesophageal Varices

Gastroesophageal variceal hemorrhage is a severe fatal complication of cirrhosis. Patients with NAFLD cirrhosis should be screened and managed for gastroesophageal varices according to AASLD practice guidelines [56]: (1) Patients with compensated cirrhosis (CC) without varices on screening endoscopy should have endoscopy repeated every 2 years; patients with CC with small varices on screening endoscopy should have endoscopy repeated every year; patients with CC without varices or with small varices who develop decompensation should have a repeat endoscopy when this occurs. (2) Traditional nonselective beta-blockers (NSBBs) (propranolol, nadolol, and carvedilol) or endoscopic variceal ligation (EVL) is recommended for the prevention of first variceal hemorrhage in patients with medium or large varices; NSBB is the recommended therapy for patients with high-risk small esophageal varices; the combination of NSBB and EVL is first-line therapy in the prevention of rebleeding.

### 6.3. Surveillance and Management of Hepatocellular Carcinoma

There is substantial evidence that cirrhosis is a common cause for the development of HCC [57]. Patients with NAFLD cirrhosis are at higher risk for HCC [58]. The cumulative incidence of HCC from NAFLD cirrhosis has been reported as 2.4% and 12.8% over a median follow-up of 3.2 to 7.2 years [59]. International societies recommend HCC surveillance in selected target populations, including patients with cirrhosis of any cause [60]. AASLD recommends that patients with NAFLD cirrhosis should be considered for HCC screening with ultrasound testing and with or without measurement of blood alpha-fetoprotein (AFP) levels, every 6 months [52, 61]. The treatment of HCC in patients with NAFLD cirrhosis may be referred to the AASLD practice guidelines [61]. T2DM significantly increases the risk of developing HCC [62]. Metformin and statins significantly reduce the risk of HCC among patients with diabetes [63]. Statins and metformin have been suggested as potential strategies for the primary prevention of HCC in patients with NAFLD and diabetes [60, 62].

### 6.4. Liver Transplantation

Liver transplant is an effective treatment for end-stage liver disease, with an overall one-year survival of around 91% and a three-year survival of around 80%. Survival rates up to ten years are similar for patients receiving transplants for NAFLD cirrhosis and those receiving transplants for other indications, such as HCV cirrhosis and alcoholic cirrhosis. Guidelines for liver transplantation for patients with nonalcoholic steatohepatitis recommend that the indications for liver transplantation include NASH cirrhosis or HCC [54]. Studies of posttransplant survival outcomes suggested that NASH cirrhosis is associated with higher 30-day mortality, predominantly from an increase in CVD, and that severe obesity is likely to increase postoperative and long term mortality [64]. Thus, patients should undergo preoperative assessment and management of CVD and optimization of nutritional status. Following liver transplantation for patients with NAFLD cirrhosis, NAFLD recurs in at least 1/3 of patients [65]. Reduced mobility and commonly used immunosuppression regimens place those patients at higher risks of developing obesity, diabetes, and hypertension or exacerbating these conditions if previously present. As a result, body weight, hypertension, diabetes, and hyperlipidemia should continue to be monitored and managed in posttransplant patients.

## 7. Prognosis

Studies on the prognosis of NAFLD cirrhosis were reported mostly several years ago. The 5-year survival rate of 68 patients with NASH cirrhosis was 75.2% [48]. The death of patients with NAFLD cirrhosis is caused by complications. Once cirrhosis develops, prognosis is negatively impacted, with potential development of cirrhosis complications. The 5-year occurrence rates of ascites, varices, encephalopathy, and HCC of 68 patients with NASH cirrhosis were 19.1%, 28.2%, 16.1%, and 11.3%, respectively [38]. The 10-year occurrence rates of ascites, variceal hemorrhage, encephalopathy, and HCC of 152 patients with NASH cirrhosis were 14%, 12%, 15%, and 7%, respectively [66]. Bhala compared the natural history of NAFLD cirrhosis to HCV cirrhosis and found that patients with NAFLD cirrhosis appeared to have lower rates of liver-related complications and lower rates of HCC than patients with HCV infection of a similar disease stage, cardiovascular mortality was greater in patients with NAFLD related cirrhosis, and these two groups of patients had similar overall mortality [39]. NAFLD cirrhosis is the larger proportion of cryptogenic cirrhosis. The studies on the natural history of cryptogenic cirrhosis showed that the cumulated incidence of HCC in patients with cryptogenic cirrhosis was similar to that in patients with HCV cirrhosis, and patients with cryptogenic cirrhosis have a higher risk of developing severe liver complications [67, 68]. These existing data strongly suggested that NAFLD cirrhosis has a poor prognosis.

## 8. Conclusions

NAFLD is becoming one of the leading causes of cirrhosis. Risk factors for the progression to cirrhosis in patients with NAFLD include NASH, metabolic factors, genetic polymorphisms, and older age. The clinical presentations of NAFLD cirrhosis are similar to those of cirrhosis caused by other etiology. The diagnosis of decompensated cirrhosis is relatively easy for patients with NAFLD. Liver biopsy, imaging, and scoring systems for fibrosis are important methods for the diagnosis of compensated cirrhosis in patients with NAFLD. Reducing weight, prohibiting drinking, managing other risk factors for progressing to cirrhosis, and antifibrosis are fundamental treatments. Screening, treatment, and prevention of cirrhosis complications and CVD are crucial for the management of NAFLD cirrhosis. Liver transplant is an effective treatment for end-stage liver disease in patients with NAFLD cirrhosis. The prognosis of NAFLD cirrhosis is poor. The prevention and treatment of NAFLD cirrhosis should be emphasized.

## References

[1] de la Garza R. G., Morales-Garza L. A., Martin-Estal I., Castilla-Cortazar I. (2017). Insulin-like growth factor-1 deficiency and cirrhosis establishment. *Journal of Clinical Medicine Research*.

[2] Stasi C., Silvestri C., Voller F., Cipriani F. (2015). Epidemiology of liver cirrhosis. *Journal of Clinical and Experimental Hepatology*.

[3] Abbas N., Makker J., Abbas H., Balar B. (2017). Perioperative care of patients with liver cirrhosis: a review. *Health Services Insights*.

[4] Mokdad A. A., Lopez A. D., Shahraz S. (2014). Liver cirrhosis mortality in 187 countries between 1980 and 2010: a systematic analysis. *BMC Medicine*.

[5] Safaei A., Oskouie A. A., Mohebbi S. R. (2016). Metabolomic analysis of human cirrhosis, hepatocellular carcinoma, non-alcoholic fatty liver disease and non-alcoholic steatohepatitis diseases. *Gastroenterol Hepatol Bed Bench*.

[6] Kadayifci A., Tan V., Ursell P. C., Merriman R. B., Bass N. M. (2008). Clinical and pathologic risk factors for atherosclerosis in cirrhosis: A comparison between NASH-related cirrhosis and cirrhosis due to other aetiologies. *Journal of Hepatology*.

[7] van den Berg E. H., Douwes R. M., de Meijer V. E., Schreuder T. C., Blokzijl H. (2018). Liver transplantation for NASH cirrhosis is not performed at the expense of major post-operative morbidity. *Digestive and Liver Disease*.

[8] Wree A., Broderick L., Canbay A., Hoffman H. M., Feldstein A. E. (2013). From NAFLD to NASH to cirrhosis-new insights into disease mechanisms. *Nature Reviews Gastroenterology & Hepatology*.

[9] Younossi Z. M., Koenig A. B., Abdelatif D., Fazel Y., Henry L., Wymer M. (2016). Global epidemiology of nonalcoholic fatty liver disease—meta-analytic assessment of prevalence, incidence, and outcomes. *Hepatology*.

[10] Angulo P. (2010). Long-term mortality in nonalcoholic fatty liver disease: is liver histology of any prognostic significance?. *Hepatology*.

[11] Kabbany M. N., Selvakumar P. K. C., Watt K. (2017). Prevalence of nonalcoholic steatohepatitis-associated cirrhosis in the United States: an analysis of national health and nutrition examination survey data. *American Journal of Gastroenterology*.

[12] Fung J., Lee C.-K., Chan M., Seto W.-K., Lai C.-L., Yuen M.-F. (2015). High prevalence of non-alcoholic fatty liver disease in the Chinese—results from the Hong Kong liver health census. *Liver International*.

[13] Kwok R., Choi K. C., Wong G. L.-H. (2016). Screening diabetic patients for non-alcoholic fatty liver disease with controlled attenuation parameter and liver stiffness measurements: a prospective cohort study. *Gut*.

[14] Lonardo A., Nascimbeni F., Mantovani A., Targher G. (2018). Hypertension, diabetes, atherosclerosis and NASH: cause or consequence?. *Journal of Hepatology*.

[15] Xiong J., Wang J., Huang J., Sun W., Wang J., Chen D. (2015). Non-alcoholic steatohepatitis-related liver cirrhosis is increasing in China: a ten-year retrospective study. *Clinics*.

[16] Michitaka K., Nishiguchi S., Aoyagi Y., Hiasa Y., Tokumoto Y., Onji M. (2010). Etiology of liver cirrhosis in Japan: a nationwide survey. *Journal of Gastroenterology*.

[17] Karageorgos S. A., Stratakou S., Koulentaki M. (2017). Long-term change in incidence and risk factors of cirrhosis and hepatocellular carcinoma in crete, Greece: a 25-year study. *Annals of Gastroenterology*.

[18] Hsiang J. C., Bai W. W., Raos Z. (2015). Epidemiology, disease burden and outcomes of cirrhosis in a large secondary care hospital in South Auckland, New Zealand. *Internal Medicine Journal*.

[19] Holmer M., Melum E., Isoniemi H. (2018). Nonalcoholic fatty liver disease is an increasing indication for liver transplantation in the Nordic countries. *Liver International*.

[20] Doycheva I., Issa D., Watt K. D., Lopez R., Rifai G., Alkhouri N. (2018). Nonalcoholic steatohepatitis is the most rapidly increasing indication for liver transplantation in young adults in the United States. *Journal of Clinical Gastroenterology*.

[21] Parikh N. D., Marrero W. J., Wang J. (2017). Projected increase in obesity and non-alcoholic-steatohepatitis-related liver transplantation waitlist additions in the United States. *Hepatology*.

[22] Siddiqui M. S., Fuchs M., Idowu M. O. (2015). Severity of nonalcoholic fatty liver disease and progression to cirrhosis are associated with atherogenic lipoprotein profile. *Clinical Gastroenterology and Hepatology*.

[23] Marengo A., Jouness R. I. K., Bugianesi E. (2016). Progression and natural history of nonalcoholic fatty liver disease in adults. *Clinics in Liver Disease*.

[24] Singh S., Allen A. M., Wang Z., Prokop L. J., Murad M. H., Loomba R. (2014). Sa1052 fibrosis progression in nonalcoholic fatty liver versus nonalcoholic steatohepatitis: a systematic review and meta-analysis of paired-biopsy studies. *Gastroenterology*.

[25] Valenti L., Bugianesi E., Pajvani U., Targher G. (2016). Nonalcoholic fatty liver disease: cause or consequence of type 2 diabetes?. *Liver International*.

[26] Porepa L., Ray J. G., Sanchez-Romeu P., Booth G. L. (2010). Newly diagnosed diabetes mellitus as a risk factor for serious liver disease. *Canadian Medical Association Journal*.

[27] Nderitu P., Bosco C., Garmo H. (2017). The association between individual metabolic syndrome components, primary liver cancer and cirrhosis: a study in the Swedish AMORIS cohort. *International Journal of Cancer*.

[28] Anstee Q. M., Seth D., Day C. P. (2016). Genetic factors that affect risk of alcoholic and nonalcoholic fatty liver disease. *Gastroenterology*.

[29] Valenti L., Al-Serri A., Daly A. K. (2010). Homozygosity for the patatin-like phospholipase-3/adiponutrin i148m polymorphism influences liver fibrosis in patients with nonalcoholic fatty liver disease. *Hepatology*.

[30] Liu Y. L., Reeves H. L., Burt A. D. (2014). TM6SF2 rs58542926 influences hepatic fibrosis progression in patients with non-alcoholic fatty liver disease. *Nature Communications*.

[31] Frith J., Day C. P., Henderson E., Burt A. D., Newton J. L. (2009). Non-alcoholic fatty liver disease in older people. *Gerontology*.

[32] Noureddin M., Yates K. P., Vaughn I. A. (2013). Clinical and histological determinants of nonalcoholic steatohepatitis and advanced fibrosis in elderly patients. *Hepatology*.

[33] Vernon G., Baranova A., Younossi Z. M. (2011). Systematic review: the epidemiology and natural history of non-alcoholic fatty liver disease and non-alcoholic steatohepatitis in adults. *Alimentary Pharmacology & Therapeutics*.

[34] Ballestri S., Nascimbeni F., Baldelli E., Marrazzo A., Romagnoli D., Lonardo A. (2017). NAFLD as a sexual dimorphic disease: role of gender and reproductive status in the development and progression of nonalcoholic fatty liver disease and inherent cardiovascular risk. *Advances in Therapy*.

[35] Rich N. E., Oji S., Mufti A. R. (2018). Racial and ethnic disparities in nonalcoholic fatty liver disease prevalence, severity, and outcomes in the United States: a systematic review and meta-analysis. *Clinical Gastroenterology and Hepatology*.

[36] Bhadoria A. S., Kedarisetty C. K., Bihari C. (2017). Impact of family history of metabolic traits on severity of non-alcoholic steatohepatitis related cirrhosis: a cross-sectional study. *Liver International*.

[37] Hui J. M., Kench J. G., Chitturi S. (2003). Long-term outcomes of cirrhosis in nonalcoholic steatohepatitis compared with hepatitis C. *Hepatology*.

[38] Yatsuji S., Hashimoto E., Tobari M., Taniai M., Tokushige K., Shiratori K. (2009). Clinical features and outcomes of cirrhosis due to non-alcoholic steatohepatitis compared with cirrhosis caused by chronic hepatitis C. *Journal of Gastroenterology and Hepatology*.

[39] Bhala N., Angulo P., van der Poorten D. (2011). The natural history of nonalcoholic fatty liver disease with advanced fibrosis or cirrhosis: an international collaborative study. *Hepatology*.

[40] Dyson J. K., Anstee Q. M., McPherson S. (2014). Non-alcoholic fatty liver disease: a practical approach to treatment. *Frontline Gastroenterol*.

[41] Caldwell S., Argo C. (2010). The natural history of non-alcoholic fatty liver disease. *Digestive Diseases*.

[42] Brown G. T., Kleiner D. E. (2016). Histopathology of nonalcoholic fatty liver disease and nonalcoholic steatohepatitis. *Metabolism—Clinical and Experimental*.

[43] Shen F., Zheng R. D., Mi Y. Q. (2014). Controlled attenuation parameter for non-invasive assessment of hepatic steatosis in Chinese patients. *World Journal of Gastroenterology*.

[44] Laurent C. (2018). Diagnosis of non-alcoholic fatty liver disease/non-alcoholic steatohepatitis: non-invasive tests are enough. *Liver International*.

[45] Koehler E. M., Plompen E. P. C., Schouten J. N. L. (2016). Presence of diabetes mellitus and steatosis is associated with liver stiffness in a general population: the Rotterdam study. *Hepatology*.

[46] Hashemi S.-A., Alavian S.-M., Gholami-Fesharaki M. (2016). Assessment of transient elastography (FibroScan) for diagnosis of fibrosis in non-alcoholic fatty liver disease: a systematic review and meta-analysis. *Caspian Journal of Internal Medicine*.

[47] Dyson J. K., Anstee Q. M., McPherson S. (2014). Non-alcoholic fatty liver disease: a practical approach to diagnosis and staging. *Frontline Gastroenterology*.

[48] Festi D., Schiumerini R., Marasco G., Scaioli E., Pasqui F., Colecchia A. (2015). Non-invasive diagnostic approach to non-alcoholic fatty liver disease: current evidence and future perspectives. *Expert Review of Gastroenterology & Hepatology*.

[49] De Lédinghen V., Wong V. W.-S., Vergniol J. (2012). Diagnosis of liver fibrosis and cirrhosis using liver stiffness measurement: comparison between M and XL probe of FibroScan. *Journal of Hepatology*.

[50] Imajo K., Kessoku T., Honda Y. (2016). Magnetic resonance imaging more accurately classifies steatosis and fibrosis in patients with nonalcoholic fatty liver disease than transient elastography. *Gastroenterology*.

[51] Stål P. (2015). Liver fibrosis in non-alcoholic fatty liver disease—diagnostic challenge with prognostic significance. *World Journal of Gastroenterology*.

[52] Chalasani N., Younossi Z., Lavine J. E. (2018). The diagnosis and management of nonalcoholic fatty liver disease: practice guidance from the American Association for the study of liver diseases. *Hepatology*.

[53] Festi D., Schiumerini R., Marasco G., Scaioli E., Pasqui F., Colecchia A. (2015). Non-invasive diagnostic approach to non-alcoholic fatty liver disease: current evidence and future perspectives. *Expert Review of Gastroenterology & Hepatology*.

[54] Newsome P. N., Allison M. E., Andrews P. A. (2012). Guidelines for liver transplantation for patients with alcoholic steatohepatitis. *Gut*.

[55] Wattacheril J., Issa D., Sanyal A. (2018). Nonalcoholic Steatohepatitis (NASH) and hepatic fibrosis: emerging therapies. *Annual Review of Pharmacology and Toxicology*.

[56] Garcia-Tsao G., Abraldes J. G., Berzigotti A., Bosch J. (2017). Portal hypertensive bleeding in cirrhosis: risk stratification, diagnosis, and management: 2016 practice guidance by the American Association for the study of liver diseases. *Hepatology*.

[57] Than N. N., Ghazanfar A., Hodson J. (2017). Comparing clinical presentations, treatments and outcomes of hepatocellular carcinoma due to hepatitis C and non-alcoholic fatty liver disease. *QJM*.

[58] Shetty K., Chen J., Shin J., Jogunoori W., Mishra L. (2015). Pathogenesis of hepatocellular carcinoma development in non-alcoholic fatty liver disease. *Current Hepatology Reports*.

[59] Baffy G., Brunt E. M., Caldwell S. H. (2012). Hepatocellular carcinoma in non-alcoholic fatty liver disease: an emerging menace. *Journal of Hepatology*.

[60] Degasperi E., Colombo M. (2016). Distinctive features of hepatocellular carcinoma in non-alcoholic fatty liver disease. *The Lancet Gastroenterology & Hepatology*.

[61] Heimbach J. K., Kulik L. M., Finn R. S. (2018). AASLD guidelines for the treatment of hepatocellular carcinoma. *Hepatology*.

[62] Wainwright P., Scorletti E., Byrne C. D. (2017). Type 2 diabetes and hepatocellular carcinoma: risk factors and pathogenesis. *Current Diabetes Reports*.

[63] El-Serag H. B., Johnson M. L., Hachem C., Morgana R. O. (2009). Statins are associated with a reduced risk of hepatocellular carcinoma in a large cohort of patients with diabetes. *Gastroenterology*.

[64] Khan R. S., Newsome P. N. (2016). Non-alcoholic fatty liver disease and liver transplantation. *Metabolism*.

[65] Zezos P., Renner E. L. (2014). Liver transplantation and non-alcoholic fatty liver disease. *World Journal of Gastroenterology*.

[66] Sanyal A. J., Banas C., Sargeant C. (2006). Similarities and differences in outcomes of cirrhosis due to nonalcoholic steatohepatitis and hepatitis C. *Hepatology*.

[67] Ratziu V., Bonyhay L., Di Martino V. (2002). Survival, liver failure, and hepatocellular carcinoma in obesity-related cryptogenic cirrhosis. *Hepatology*.

[68] Rinaldi L., Nascimbeni F., Giordano M. (2017). Clinical features and natural history of cryptogenic cirrhosis compared to hepatitis C virus-related cirrhosis. *World Journal of Gastroenterology*.

